# Epididymal epithelial degeneration and lipid metabolism impairment account for male infertility in occludin knockout mice

**DOI:** 10.3389/fendo.2022.1069319

**Published:** 2022-11-28

**Authors:** Bao Ying Liu, Bao Li Zhang, Da Yuan Gao, Qing Li, Xin Yu Xu, Winnie Shum

**Affiliations:** ^1^ School of Life Science and Technology, Shanghai Tech University, Shanghai, China; ^2^ Center for Excellence in Molecular Cell Science, Shanghai Institute of Biochemistry and Cell Biology, Chinese Academy of Sciences, Shanghai, China; ^3^ University of Chinese Academy of Sciences, Beijing, China; ^4^ National Health Commission (NHC) Key Lab of Reproduction Regulation, Shanghai Institute for Biomedical and Pharmaceutical Technologies, Fudan University, Shanghai, China

**Keywords:** occludin, arachidonic acid, V-ATPase, lipid metabolism, luminal acidification, epididymis

## Abstract

Occludin (OCLN) is a tight junction protein and Ocln deletion mutation causes male infertility in mice. However, the role of OCLN in male reproductive system remains unknown. In this study, we used an interdisciplinary approach to elucidate the underlying mechanism of male infertility in related to OCLN function, including *Ocln* knockout mice as well as a combined omics analysis and immunofluorescent labelling. Our results showed that the epididymis of *Ocln*-null mice displayed a phenomenon resembling epididymal sperm granuloma, which occurred especially in the junctional region between caput and corpus epididymidis. Sperm motility and fertilisation capacity were also impaired in these *Ocln*-null mice, accompanied by enlarged tubules in the proximal regions and degeneration in the distal regions of epididymis. Cellular localization analysis showed that OCLN immunofluorescence was enriched only in the apical junction of epithelial principal cells in the proximal regions of epididymis. Integrative omics analysis revealed the downregulation of gene clusters enriched in acid secretion and fatty acid metabolism in the *Ocln*-null epididymis, especially the enzymes related to the unsaturated arachidonic acid pathway. The number of proton-pump V-ATPase-expression clear cells, a key player of luminal acidification in the epididymis, declined drastically from prepubertal age before sperm arrival but not in the early postnatal age. This was accompanied by programmed cell death of clear cells and increased pH in the epididymal fluid of OCLN-deficient mice. The lipidomics results showed significantly increased levels of specific DAGs conjugated to unsaturated fatty acids in the Ocln-mutant. Immunofluorescent labelling showed that the arachidonic acid converting enzyme PTGDS and phospholipase PLA2g12a were prominently altered in the principal cells and luminal contents of the *Ocln*-mutant epididymis. Whereas the carboxylate ester lipase CES1, originally enriched in the WT basal cells, was found upregulated in the *Ocln*-mutant principal cells. Overall, this study demonstrates that OCLN is essential for maintaining caput-to-corpus epithelial integrity, survival of acid-secreting clear cells, and unsaturated fatty acid catabolism in the mouse epididymis, thereby ensuring sperm maturation and male fertility.

## Introduction

The epididymis is a post-testicular male reproductive organ to provide a region-specific and optimal luminal microenvironment for sperm maturation and storage before sperm transport to the vas deferens for ejaculation (1–3). Such epididymal microenvironment is maintained by the epithelium lining the long and highly convoluted epididymal duct, which consists of several epithelial subtypes, including basal, clear and principal epithelial cells (4–8). Although sperm acquire a degree of motility during epididymal transit, they remain quiescent in the epididymis, which is believed to be achieved by the slightly acidic (pH 5.5-6.8) (5, 9) luminal environment built by epididymal epithelial cells. It is known that bicarbonate ( ${\text{HCO}}_{3}^{-}$ )-rich alkaline environment, such as in the uterine cavity fluid, triggers the sperm to undergo hyperactivation and final stage of capacitation (10–13), and premature activation can be resulted (14). Thus, such ${\text{HCO}}_{3}^{-}$ rich alkaline environment is detrimental to sperm storage in the epididymis. This is also evident by that impaired luminal acidification or increased bicarbonate is known to contribute to male infertility (1, 7, 8, 15, 16). In addition, the viability of the numerous sperm cells in the lumen is also supported by the reciprocal exchanges of nutrients and metabolic materials, such as membrane-containing debris, lipids and proteins (1, 4–8, 17). Moreover, lipid homeostasis is particularly essential for sperm maturation and defects of lipid metabolism are associated with sperm impairment, male infertility, and metabolic syndromes (17–22). All these physiological processes are maintained by the intriguing intercellular communication network between sperm cells and epididymal epithelium.

Occludin (OCLN) is an epithelial tight junction protein and *Ocln* knockout (*Ocln-*KO) male mice are infertile (23); however, its physiological role and the mechanism underlying male infertility have not been known. The present study investigated the role of OCLN in sperm maturation in epididymis by determining its role in luminal microenvironment homeostatic regulation. The dysfunction of epididymis in the *Ocln-*KO infertile male mice was first confirmed. Attempts were then made to correlate the epididymal function with the differential expression genes (DEGs) in the transcriptomes and proteomes of *Ocln-*KO compared to WT mice. The cellular expression of the significantly downregulated DEGs, including proton-pump V-ATPase and fatty acid catabolic enzymes PTGDs and CES1, were found to be altered in specific epithelial cell types. Overall, our study demonstrated the important role of OCLN in acid-base balance and metabolism in the epididymis.

## Materials and methods

### Animals

*Ocln*-KO and B1-V-ATPase-EGFP mice were provided by Prof. Eveline E. Schneeberger and Prof. Sylvie Breton from MGH/Harvard Medical School. The background of these mice has been published previously (23, 24). All animals were housed in a standard specific pathogen-free animal house. Since male *Ocln*-KO mice are sterile, these mice were produced from heterozygous pairings; otherwise, mice were bred with wild-type (WT) mice (C57BL/6) for maintenance. All animal experiments were approved by the Institutional Animal Care and Use Committee (IACUC) of ShanghaiTech University.

### Fertility test

WT and *Ocln*-KO male mice aged 6, 9, 12, 20, 40 and 60 weeks were mated with two female wild-type mice. At least three pairs of mice of each age were mated for at least two weeks. To determine male mice mating behavior, the female mice (12-week-old) from the mating cage were examined for the plugs and the vaginal plugs were smeared, and the spermatozoa were observed under microscopy.

### IVF, ICSI, and CASA assays

For *in vitro* fertilization (IVF) and intracytoplasmic sperm injection (ICSI) assays, sperms were directly obtained from caput or cauda epididymis of WT and *Ocln*-KO males, the assays were performed as previously reported (25, 26). For computer-assisted sperm analysis (CASA) assays, sperm from the cauda or caput epididymidis were incubated in the EKRB capacitation medium and analyzed using an HTM-TOX IVOS sperm motility analyzer (Hamilton-Thon Research, v14), as we published previously (25, 27).

### Hematoxylin and eosin (HE) staining

The testes and epididymides of WT and *Ocln*-KO male mice were fixed with paraformaldehyde-lysine-periodate (PLP), embedded in O.C.T. compound (Sakura) and sectioned at −18°C or −20°C (Leica Microsystems GmbH), as described previously (28, 29). Cryosections were rehydrated in PBS and stained with Hematoxylin and Eosin Staining kit (Beyotime). Images were acquired using the slide scanner Olympus VS120.

### Immunofluorescent labelling and TUNEL assay

The immunofluorescent labelling procedure was performed as described previously (25). Briefly, Cryosections of testes and epididymides tissue of WT and *Ocln*-KO male mice were rehydrated in PBS, antigen retrieved with 1%w/v SDS, blocked with 1% w/v BSA and then incubated with primary antibodies, followed by incubated with the secondary antibodies. All slides were mounted in Vectashield with DAPI. The TUNEL assay was performed as the manual of Click-iT Plus TUNEL Assay Kit (Invitrogen). Images were acquired using laser scanning confocal microscopy (LSM880, Carl Zeiss or Nikon A1R) and analyzed with the original software before imported into Adobe Photoshop for typesetting. Details of the antibodies used in this study are listed in **Supplementary Table 1**.

### Western blot

Western blot was performed as published previously (27). Cell lysates were subjected to SDS-PAGE and then transferred to PVDF membranes. The membranes were immunoblotted with the indicated antibodies and visualized by SuperSignal™ West Pico PLUS Chemiluminescent Substrate (Thermo Scientific). Data were acquired using an Amersham Imager 600 or 680 system (GE Healthcare, NJ, USA). Antibodies details are listed in **Supplementary Table 1**.

### Cell cultures and *Ocln*-mutant cell lines

DC2 cell lines were cultured in an incubator at 33°C, 5% CO_2_ with Full-IMDM supplemented with 1 nM 5α-dihydrotestosterone and containing 10% (v/v) FBS, penicillin and streptomycin, as described previously (30). *Ocln*-KO cells were generated in DC2 cells by using the CRISPR/Cas9 system. The *Ocln* sequence in the second exon 5′-CGGCTGAGAGAGCATCGGC-3′ (*Ocln*–sgRNA) was targeted with pGL3-U6-sgRNA-PGK-Puro (Addgene). DC2 cells were then co-transfected with *Ocln*–sgRNA-expressing vector and Cas9 expressed vector pST1374-NLS-flag-linker-Cas9 (Addgene) by Lipofectamine 2000 and selected with puromycin and blasticidin for 24  h. Single cells were isolated by cell sorting. The single clones were validated by immunoblotting analysis and DNA sequencing. The two plasmids were gifts from Xingxu Huang (31, 32).

### pH measurement of vas deferens luminal fluid

For pH measurement in vas deferens of adult WT and *Ocln*-KO male mice, the animals were anesthetized and both sides of the vas deferens were dissected, the blood was removed and kept the vas deferens under a fat pad. The luminal liquid in the sectioned vas deferens was squeezed out and dropped on the pH paper (Hydrion, pH range 5.5-8.0) and recorded it by video. Data values were measured on captured images 10- second after the sample liquid touched the pH paper and the different standards in grey scale were used for calibration using Fiji software.

### Lipid extraction and lipidomics analysis

For lipid extraction, the proximal epididymides from three WT or *Ocln*-KO mice were pooled into one sample. The distal epididymidum was treated the same way. Three samples per group were used in this study. Lipids were extracted using a modified version of the Bligh and Dyer’s method as described previously (33). Lipidomic analyses were conducted at LipidALL Technologies using an Agilent 1290 II UPLC coupled with Sciex QTRAP 6500 PLUS as reported previously (34). Glycerol lipids including diacylglycerols (DAG) and triacylglycerols (TAG) were quantified using a modified version of reverse phase HPLC/MRM. Heatmaps and volcano plots of TAG and DAG analyses were performed on the free online data analysis website Tutools platform (https://www.cloudtutu.com/).

### Proteomics analysis using tandem mass tags (TMTs) labelling method

The protein preparation and TMTs labelling workflow have been described previously (35). Briefly, total proteins were extracted from three pairs of WT and *Ocln*-KO mice whole epididymis by lysis buffer (50 mM (NH_4_)_2_CO_3_, 8 M urea, 1 mM DTT and protease inhibitor) separately and quantitation using BCA protein assay kit. After alkylated by iodoacetamide and digested by trypsin, the peptide was labeled by TMTs label regent following the manufacturer’s protocol. The resulting peptides were separated and analyzed on an Easy-nLC 1000 system coupled to a Q Exactive HF (Thermo Scientific). The LC-MS/MS procedure and data analysis were performed as reported previously (36).

### RNA-seq analysis

Total RNA samples of whole epididymis were extracted from three pairs of WT and *Ocln*-KO mice by Trizol reagent separately according to the manufacturer’s instructions. The RNA quality was checked by Bioanalyzer 2200 (Agilent) and kept at -80°C.The RNA with RIN >6.0 is about to undergo rRNA depletion. cDNA libraries were constructed for each pooled RNA sample by using the NEBNext^®^ Ultra™ Directional RNA Library Prep Kit for Illumina according to the manufacturer’s instructions. The tagged cDNA libraries were pooled in equal ratio and used for 150 bp paired-end sequencing in a single lane of the Illumina HiSeqXTen. Before read mapping of pair-ends, clean reads were obtained from the raw reads by removing the adaptor sequences, reads with >5% ambiguous bases (noted as N) and low-quality reads containing more than 20 percent of bases with qualities of<20. The clean reads were then aligned to mouse genome (version: GRCm38 NCBI) using hisat2. Differential gene and transcript expression analysis of RNA-seq experiments with HTseq (37) was used to count mRNA, and the RPKM method was used to determine gene expression.

### Bioinformatics analysis

For RNA-Seq data, we applied the DESeq2 algorithm (38) to filter the differentially expressed genes (DEGs); for significance analysis, *P* values and false discovery rate (FDR) analysis were obtained from the following criteria (39): mRNA under the following criteria: i) Fold change > 2 or< 0.5; ii) FDR< 0.05. For proteomic data, two-tailed student’s t-test was used to verify the significance of the differences between each comparison, proteins with the fold change > 1.2 or< 0.833 and *P*-value< 0.05 were chosen.

### Quantification and statistical analysis

Comparisons were performed using the 2-tailed paired or unpaired Student’s t-test for only two groups, or one-way or 2-way-ANOVA for the comparisons of more than two groups at one- or two-levels, respectively. Significance was defined as a *P*-value< 0.05. Prism 8.0 (GraphPad) was used for all statistical analyses.

## Results

### Epididymal defects account for male infertility in *Ocln-*KO mice

**Figure 1:**
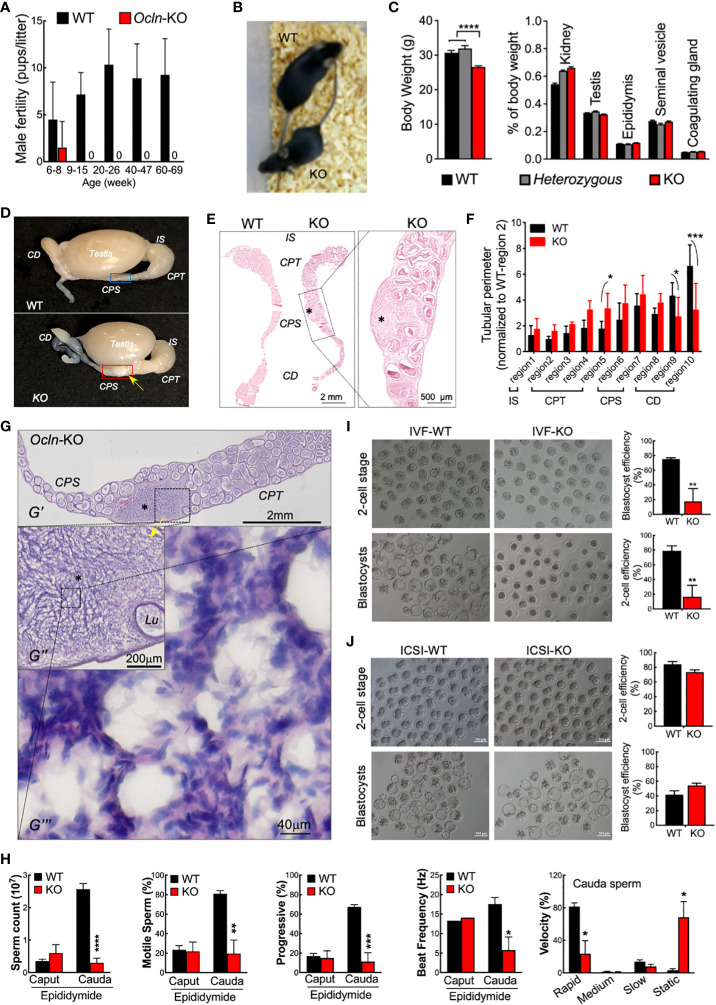
Dysfunction epididymal function rather than testicular spermatogenesis underlies the male infertile in OCLN-deficient mice. **(A)** Fertility test confirmed infertility of adult but not juvenile *Ocln-*KO male mice. **(B)** Appearance, body weights and relative organ weights **(C)** of WT, heterozygous and *Ocln-*KO male mice. **(D)** Gross anatomy showing granuloma-like tissue knot (yellow arrow) in *Ocln-*KO but not in WT epididymis. **(E)** Alizarin red S staining showed enlarged CPS but degenerated CD epididymidis of *Ocln-*KO mice compared with WT. Asterisks: granuloma-like structure in the *Ocln-*KO CPT-CPS conjunctions. **(F)** Regional tubular perimeters of epididymis of WT and *Ocln-*KO epididymis. Annotations of the different epididymal segments were as described previously (40), *viz.* region 1 for initial segment (IS), region 2-5 for caput (CPT), region 6-7 for corpus (CPS), and region 8-10 for cauda (CD) epididymidis. **(G)** H&E staining showing granuloma-like tissue knot (asterisks) in the CPT- CPS junction of an *Ocln-*KO epididymal cryosection (*G’*). Intermediate resolution image showing the enlarged interstitial space filled with many sperm next to the distorted tubule (*Lu*) (*G’’*). Higher resolution image showing that numerous sperm, characterized by their oval-shaped head (purple) and curved tail (pink), essentially filling the space of the enlarged interstitial tissue (*G’’’*). **(H)** Decreased sperm counting of cauda sperm but not caput sperm and CASA parameters showing impaired motility of *Ocln-*KO cauda sperm. **(I)** IVF **(I)** and ICSI **(J)** assay results using cauda epididymidal spermatozoa of WT or *Ocln-*KO mice. Data were means ± SD from (n > 3 animals per group). *P<0.05, **P<0.01, ***P<0.001, ****P<0.0001, one- or two-way ANOVA.

*Ocln-*KO male mice were reported to be infertile (23). We also monitored the fertility of the *Ocln-*KO male mice over a long period (from 6-week-old till 69-week-old), which confirmed their sterility after 9-week-old. However, at juvenile ages of about 8 weeks, 1 out 8 *Ocln-*KO male mice was able to fertilize female mice to give birth to live pups (**Figure 1A**), suggesting the male sterility possibly an acquired phenotype as the mice grow. We then investigated if the male infertility could be due to problems of the epididymis. The *Ocln-*KO males had smaller overall in size compared to the wild-type (WT) mice, although no difference in proportional size of the epididymis or other organs was found (**Figures 1B, C**). Through detailed morphological analysis, we frequently found the testis of the *Ocln-*KO was morphological normal (**Supplementary Figures S1A-C**). Whereas the caput (CPT) epididymidis in the head portion of epididymis was enlarged and cauda (CD) epididymidis in the tail portion was degenerated in the *Ocln-*KO mice as compared to those of the WT (**Figures 1D-F**). Strikingly, in caput to corpus (CPT-CPS) junctional segments of the *Ocln-*KO, sperm cells were found infiltrated into the interstitial area causing tissue enlargement in this region (**Figure 1G**), a pathology similar to epididymal sperm granulomas (41, 42).

We next examined the epididymal sperm from *Ocln-*KO mice in comparison with the WT. CASA showed that CPT sperm from the epididymal head were mostly quiescent in both WT and *Ocln-*KO mice, although CD sperm from the epididymal tail of WT mice gained substantially higher motility and progressiveness as compared to the CPT sperm (**Figure 1H**). However, such a CPT to CD increase in sperm motility was totally absent in *Ocln-*KO mice. In addition, in WT mice, the sperm count increased from CPT to CD suggesting sperm condensation through epididymal transit. Whereas, in the *Ocln-*KO mice, the CD sperm count was found at a very low level even lower than their CPT sperm count. Consistently, although the *Ocln-*KO mice copulated normally with WT females, next-morning analyses of virginal plugs of WT females after copulation revealed very few sperm, compared to control male mice (**Supplementary Figure S1D**). We next took the CD sperm for analyzing their fertilizing capacities *in vitro*. The IVF assay by incubating CD sperm with WT eggs showed significant reduction in the efficiency of *Ocln-*KO CD sperm to generate two-cell or blastocyst stage of embryos, as compared to that of WT CD sperm (**Figure 1I**). However, when we injected WT eggs with *Ocln-*KO CD spermatozoa by ICSI, the eggs were successfully activated and fertilized at a similar rate compared to WT CD spermatozoa (**Figure 1J**). After transplantation in the female oviduct, the ICSI fertilized eggs with WT of *Ocln-*KO sperm developed normally to birth (**Supplementary Figures S1E, F**), suggesting that the *Ocln-*KO CD sperm were genetically fine. All these observations suggest that male infertility in *Ocln-*KO mice is largely due to epididymal-dependent impairment of sperm transportation and thereby motility and fertilization capacity.

**Figure 2:**
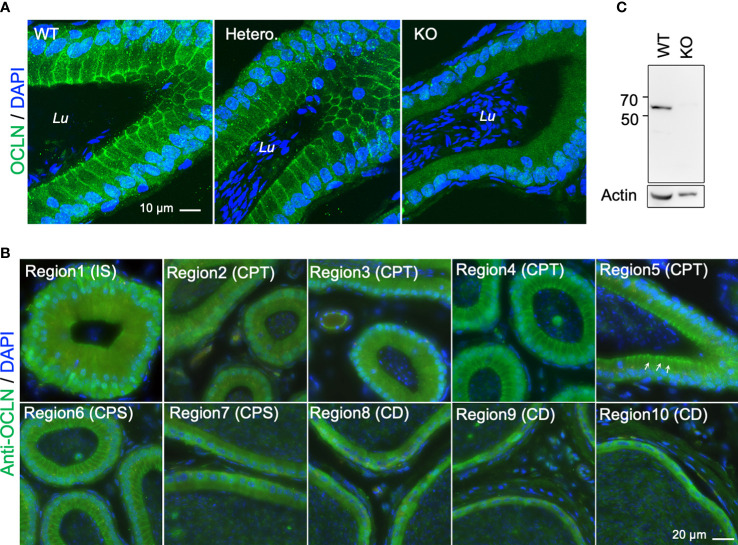
**(A)** Typical immunofluorescent-labelling of caput epididymal cryosection immunostaining for anti-OCLN antibody showing enriched OCLN protein in the apical junctional membranes of principal cells in epididymis from WT and heterozygous mice, but absent in *Ocln*-KO mice. **(B)** Immunofluorescent-labelling of epididymal cryosection immunostaining for anti-OCLN antibody showing enriched OCLN in region1-6 (IS, CPT and proximal CPS (pCPS) epididymidis). In IS, OCLN is only present in the TJs of principal cells. In the CPT and pCPS principal cells, OCLN also presents in the apical paracellular membranes (arrows). **(C)** WB-analysis of the same anti-OCLN antibody with KO and WT DC2 cell lysates. Lu, lumen; IS, initial segment; pCPT and dCPT, proximal and distal caput; CPS, corpus; CD, cauda epididymidis; Blue, DNA stained with DAPI.

To further determine the role of OCLN in the epididymis, we investigated the cellular localization of OCLN on the epididymal cryosections of WT adult mice. The immunofluorescent staining with an anti-OCLN antibody revealed that OCLN protein was enriched in the tight junctions (TJs) of principal cells of the initial segment (IS) and CPT epididymidis, as well as the lateral membranes of WT or *Ocln*-heterozygous CPT principal cells (**Figure 2A**), but hardly detectable in the distal corpus (dCPS) and CD epididymidis in the tail region of epididymis (**Figure 2B**). No specific OCLN immunofluorescence was detected in *Ocln-*KO CPT principal cells, confirming the anti-OCLN antibody specificity. Using the same anti-OCLN antibody, only one band was detected in the WT DC2 epididymal cells with a size corresponding to OCLN protein, which was significantly diminished in the *Ocln-*mutant DC2 cells (**Figure 2C**).

### Transcriptomics and proteomics revealed downregulation of acid secretion and metabolic pathways in the OCLN*-*deficient epididymis

**Figure 3:**
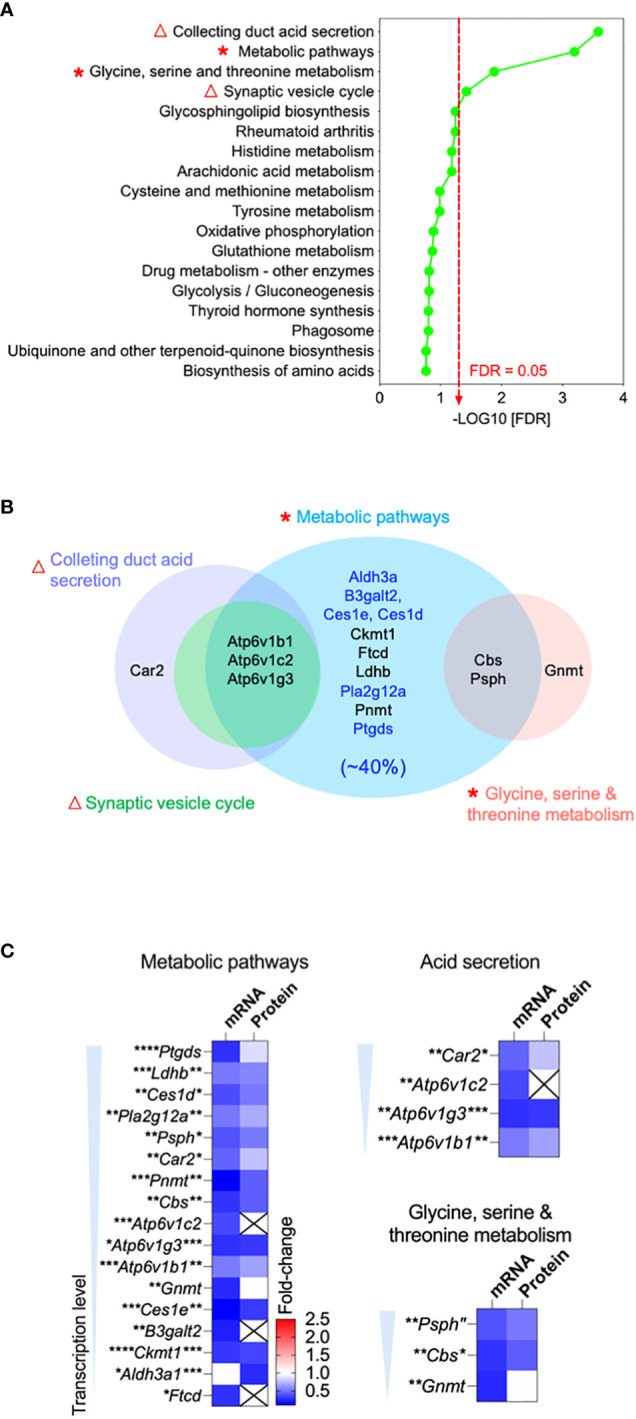
Bioinformatics revealed downregulation of acid secretion and metabolic pathways in the OCLN-deficient epididymis. **(A)** Integrative bioinformatic analysis revealed the downregulated acid secretion (triangles) and metabolic pathways (asterisks) in the epididymis of OCLN-deficient versus WT mice. Plot showing the enriched KEGG pathway terms obtained using all differential expressed genes (DEGs) of the combined transcriptomic and proteomics of whole epididymis of adult OCLN*-*deficient versus WT mice. Triangles indicate the impaired acid secretion associated pathways and asterisks metabolic associated pathways. **(B)** Venn diagram of the shortlisted DEGs in the significantly downregulated metabolic pathways in panel **(A)** Blue terms indicate the enriched genes for the enzymes relating lipid catabolism pathway, which makes up 40% of the shortlisted genes. **(C)** Heatmaps showing the fold-changes of transcriptional and proteomic levels of the shortlisted DEGs in the significantly altered pathways, including metabolism, acid secretion and amino acid metabolism. Data were means ± SD from (n > 3 animals per group). *P<0.05, **P<0.01, ***P<0.001, ****P<0.0001, unpaired t-test.

To explore how *Ocln*-KO leads to the observed epididymal defected, we profiled the transcriptomes and proteomes of the whole epididymis from adult *Ocln-*KO mice in comparison to those of WT mice to find differentially expressed genes (DEGs). An integrative approach was applied to the analysis of DEGs obtained by combining RNAseq and proteomic datasets. Through the Kyoto Encyclopedia of Genes and Genomes (KEGG) analysis, the results showed that the gene clusters enriched in the acid secretion, and metabolic pathways, including lipid and amino acid metabolic pathways, were the main nodes of downregulation (**Figure 3A**, asterisks and triangles). In addition, the significantly downregulated pathways in the *Ocln-*KO epididymis, including synaptic vesicle cycle and collecting duct acid secretion (**Figure 3A**, triangles), also implied the impairment in trafficking of proton pump V-ATPases, which are highly expressed in clear cells and responsible for the luminal acidification of epididymis and maintaining sperm in a quiescent stage (43).

Bioinformatic analysis using Venn diagram of the shortlisted DEGs with significantly downregulated pathways revealed that almost all of them were metabolic enzymes, of which about 40% were involved in lipid catabolic processes (**Figure 3B**). Heatmaps of metabolically clustered genes showed that top enzymes were involved in lipid catabolism (**Figure 3C**). Two of the top shortlisted enzymes *Ptgds* and *Pla2g12a* implicated in the metabolism of the fatty acid arachidonic acid. Another top-listed enzyme was CES1, which is also known as a lipase of carboxylic esters to release carboxylic acids and free fatty acids (FFA), such as arachidonic acid (44, 45). *Ldhb* relating to pyruvate metabolism was also a top shortlisted enzyme, while *Psph* and *Cbs* were shortlisted in the amino acid metabolism cluster. Another cluster of top listed genes were the subunits of proton-pump V-ATPase family, such as B1-V-ATPase (*Atp6v1b1*), a key player in luminal acidification of epididymis, keeping sperm in a quiescent stage (5, 46, 47).

### Increased luminal pH in distal epididymis due to the loss of proton-pump expressing clear cells in OCLN-deficient mice

**Figure 4:**
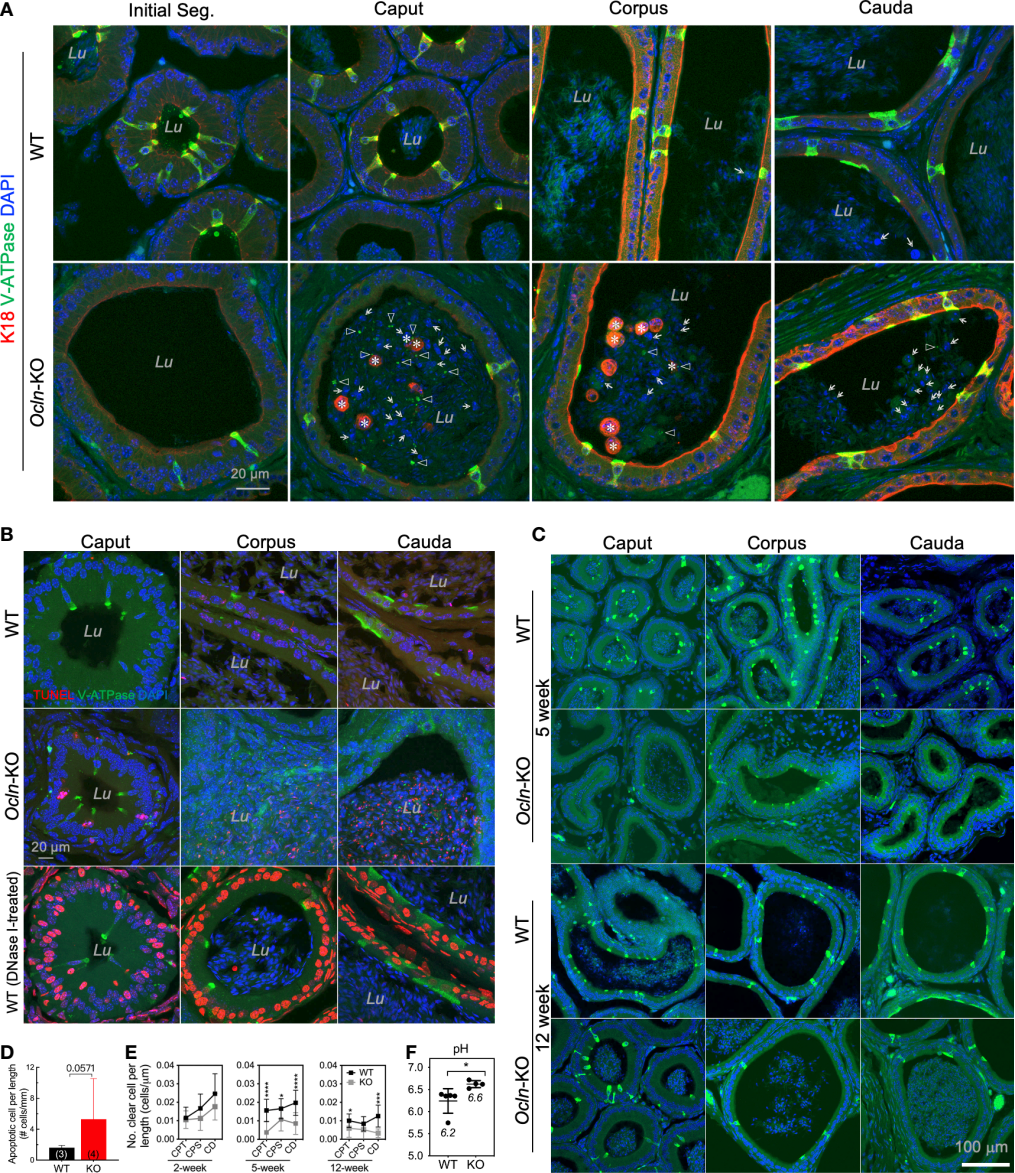
Increased luminal pH in distal epididymis and the loss of proton-pump expressing clear cells in the OCLN-deficient mice. **(A)** Immunofluorescent staining of epididymal cryosections for clear cell marker B1-V-ATPase and epithelial marker intermediate filament keratin-18 (K18) showing increased shed epithelial cells in the epididymal lumen of *Ocln-*KO (*Ocln^-/-^*) compared to WT (*Ocln^+/+^*) mice. Lu: lumen. Triangles: B1-V-ATPase-positive cell debris in the lumen. Asterisks: keratin-18-positive epithelial cells (both principal cells and clear cells) shed into the lumen. Arrows: keratin-18-negative cells in the lumen. **(B)** TUNEL staining for the different regions of epididymis from WT and *Ocln-*KO mice showing increased apoptotic index in the distal epididymal lumen (*Lu*). **(C)** Representative immunofluorescent staining images of epididymal cryosections for clear cell marker B1-V-ATPase (green) confirmed the decreased numbers of clears in all the regions of epididymis from *Ocln-*KO mice, compared to WT mice. **(D)** Quantitative TUNEL-positive epithelial cells in the epithelium and in the lumen of epididymis of WT (n = 3) and *Ocln-*KO (n = 4) mice. **(E)** Quantification of the numbers of clear cells per epithelial length statistic in WT and *Ocln-*KO mouse epididymis during development. Data represent means ± SD (n ≥ 3 animals per group, two-way ANOVA *P<0.05, ***P<0.001, ****P<0.0001). CPT, caput; CPS, corpus; CD, cauda epididymidis. **(F)** Increased pH detection in the luminal fluid of vas deferens in *Ocln-*KO mice compared with WT mice. Two-tailed unpaired t-test, *P<0.05 (n>4 animals per group).

Given the observed downregulation of V-ATPase genes in the *Ocln-*KO epididymis, we analyzed the cellular localization pattern of clear cells using V-ATPase as the marker. Our results showed that, in the epididymis of *Ocln-*KO compared to WT mice, not only the V-ATPase-positive clear cells and their cellular debris were found in the epididymal lumen, especially in the caput region. In addition, many keratin-18-positive epithelial cells were shed into the lumen of caput epididymidis (**Figure 4A**). Using TUNEL staining, we observed increased apoptotic index in *Ocln-*KO epididymis compared to WT controls, including some epithelial cells of the epididymal mucosa and some somatic cellular DNA fragments in the lumen (**Figure 4B**). In the TUNEL positive control wildtype epididymis cryosections treated with DNase I to induce DNA breaks in genome, all the somatic cells showed labelling for TUNEL. Notably, DNase I-induced DNA fragmentations did not occur in sperm cells, suggesting the DNA in sperm cells is insensitive to DNase I and well-protected from somatic enzymatic challenges.

To determine whether the loss of clear cells might be due to dysfunctional differentiation during postnatal development, in addition to increased programmed cell death during epithelial homeostasis, we harvested epididymal tissue from different ages and labelled them for the clear cell marker B1-V-ATPase. Our results showed that the number of clear cells of 2-week-old KO epididymides was only mildly different from that of WT (not shown), but it obviously decreased at puberty and in adulthood (**Figure 4C**). A trend towards increased TUNEL-positive apoptotic epithelial cells was observed in the lumen and epithelium of the *Ocln-*KO epididymis compared to WT, although statistics showed no difference (**Figure 4D**, *P*<0.06). In the luminal contents, the apoptotic index was decreased from CPT to CD epididymidis, whereas the apoptotic index was increased in the epithelial cells of epithelium (**Supplementary Figure S2**). The co-localization of TUNEL and B1-V-ATPase was observed only in some but not all B1-V-ATPase cellular debris in the luminal contents of caput epididymal, whereas some B1-V-ATPase-positive cells were not positive for TUNEL (**Supplementary Figure S2**). Quantification results showed that the numbers of V-ATPase-labelled clear cells in the epididymis were prominently decreased in the pubertal and adult mice but not statistically in early age of *Ocln*-KO mice (**Figure 4E**).

The acidity of luminal fluid of proximal vas deferens—an organ connecting successively to caudal epididymis with the presence of abundant clear cells—was also significantly decreased, suggesting a disorder of epididymal luminal microenvironment after *Ocln* deletion. Consistently, the luminal pH of *Ocln-*KO group was significantly higher than that of WT group (**Figure 4F**).

### Heterogeneous lipid content in the proximal versus distal regions of epididymis in WT mice

**Figure 5:**
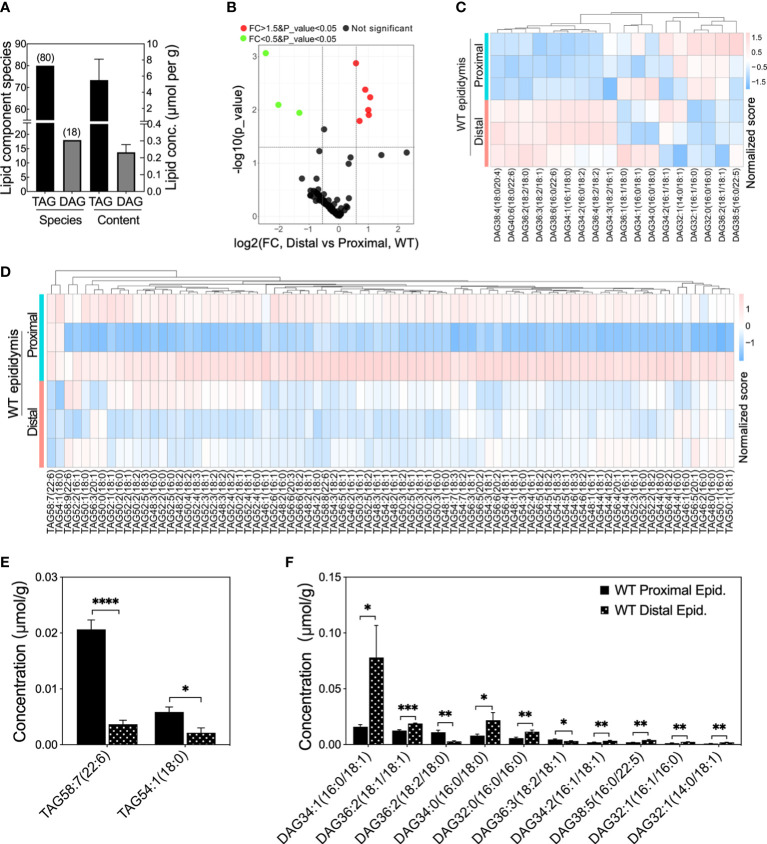
Lipid compositions at different levels in proximal and distal epididymis of WT mice. **(A)** Lipid compositions and contents detected in mouse epididymis. **(B)** Volcano plot of fold change (FC) of TAG and DAG component concentrations detected in the distal versus proximal epididymis of WT mice. Proximal epididymis annotated the samples from regions 1 to 6 whereas distal epididymis annotated the regions from 7 to 10 (n > 3 animals per group). Green dots represent specific lipid concentrations significantly less than 0.5 fold changes (FC) and red dots for those 1.5 folds greater, *P*<0.05 for both; black dots represent those lipid components have no difference in lipid concentrations. **(C)** Heatmap showing the row-normalized contents of DAG components in the proximal and distal epididymis of WT mice. **(D)** Heatmap showing the variations of the row-normalized contents of TAG components in the proximal and distal epididymis of WT mice. **(E)** Significantly lower levels of specific TAG components and prominent heterogeneous levels of DAG components **(F)** in the distal epididymis compared to the proximal epididymis of WT mice. Data were means ± SD, two-tailed unpaired *t-*test, *P< 0.05, **P< 0.01, ***P< 0.001, ****P< 0.001 (n > 3 experiments per group).

To determine whether the fatty acid homeostasis is disrupted after *Ocln* deletion, we determined the contents of stable fatty acid pools triacylglycerols (TAG) and their derivatives diacylglycerols (DAG) in the epididymis (**Figure 5** and **Supplementary Figure 3**). The lipidomic results showed that total of eighty species of TAG components and eighteen DAG components were determined in the mouse epididymis (**Figure 5A**). The total mean TAG in the mouse whole epididymis was ~5.5 µmol per gram of tissue while DAG was ~0.23 µmol per gram of tissue (**Figure 5A**). The volcano plot revealed that only a few specific DAG and TAG components had significantly different levels in the proximal versus distal epididymis (**Figure 5B**). Heatmap analysis showed the heterogeneous levels of specific DAG and TAG components in the proximal and distal epididymis of WT mice (**Figures 5C, D**). Regarding TAG, only two species showed statistically lower levels in the distal epididymis compared to the proximal epididymis, including TAG containing double-bonded unsaturated long-chain fatty acids (**Figure 5E**). As for DAG, most of the statistically distinct components had higher levels in the distal compared to the proximal epididymis of WT mice, including the DAG with double-bonded unsaturated long-chain fatty acids (**Figure 5F**).

### Disturbed acylglycerolipid homeostasis in the OCLN-deficient epididymis

**Figure 6:**
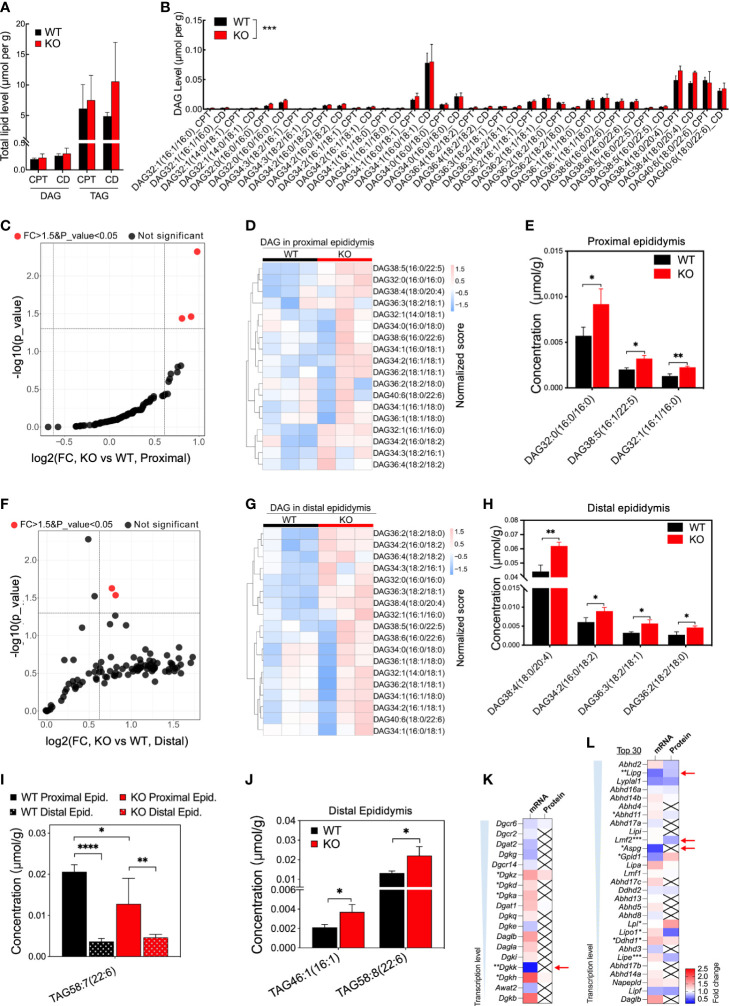
Increased concentrations of specific DAG components and downregulated transcription of enzymes in *Ocln-*KO epididymis. **(A)** No difference in the total contents of DAG and TAG compositions detected in the proximal and distal epididymis of WT and *Ocln-*KO mice. **(B)** Higher levels of specific DAG components in the proximal and distal epididymis of *Ocln-*KO mice compared to WT mice. **(C)** Volcano plot of DAG components in the proximal epididymis of *Ocln-*KO compared to WT mice. Red dots represent components with FC greater than 1.5 and significant differences at a P-value less than 0.5. Black dots represent no difference components. **(D)** Heatmap showing the content variation of DAG compositions in the proximal epididymis of *Ocln-*KO and WT mice. **(E)** Specific DAGs at higher contents in the proximal epididymis of *Ocln-*KO versus WT mice. **(F)** Volcano plot of DAG components in the distal epididymis of *Ocln-*KO and WT mice. **(G)** Heatmap showing the content variation of DAG compositions in the distal epididymis of *Ocln-*KO compared to WT mice. **(H)** Specific DAGs at higher contents in the distal epididymis of *Ocln-*KO versus WT mice. **(I)** Patterns of the specific TAG components showing significantly different levels in the *Ocln-*KO versus WT epididymis. **(J)** Significantly increased TAG components in the distal epididymis of *Ocln-*KO versus WT mice. **(K)** Heatmaps of the DEGs relating to DAG converting enzymes, including DAG kinases **(K)** and lipases **(L)** in the transcriptomes and proteomes of whole mouse epididymis. Data were means ± SD, two-way ANOVA in A, B and I, two-tailed unpair t-test in F, J-L; *P< 0.05 and **P< 0.01, ***P< 0.001, ****P< 0.0001 (n>3 experiments per group).

In the epididymis of *Ocln-*KO versus WT mice, the total mean contents of diacylglycerols (DAGs) and triacylglycerols (TAGs) did not differ between the two groups (**Figure 6A**). When all DAG components of proximal and distal epididymis were compared, respectively, the DAG components were overall significantly different in *Ocln-*KO epididymis from that of WT mice (**Figure 6B**). In the proximal epididymis, three DAG components were significantly increased, including the DAG containing double-bonded fatty acid DAG38:5(16:0/22:5) (**Figures 6C-E**). Four kinds of DAG components were significantly increased in the distal epididymis, including the most accumulated species DAG38:4(18:0/20:4), which contains the double-bonded 20-carbon arachidonic fatty acid chain (**Figures 6F-H**). For the TAG, one component with double-bonded long fatty acid chain, TAG58:7(22:6), was significantly decreased in the KO compared to WT epididymis and between proximal and distal epididymis of KO epididymis (**Figure 6I**). Whereas in the distal epididymis of KO mice, the double-bonded long fatty acid chain containing TAG components, TAG46:1(16:1) and TAG58:8(22:6), were significantly increased (**Figure 6J** and **Supplementary Figure 4**). Expression analysis in the transcriptomes and proteomes showed only slight changes in the expression of DAG kinases (**Figure 6K**) and moderate downregulation of a few lipases of DAG or TAG (**Figure 6L**).

### Altered cellular localization of the fatty acid catabolic enzymes CES1 and PTGDS in the OCLN-deficient epididymis

**Figure 7:**
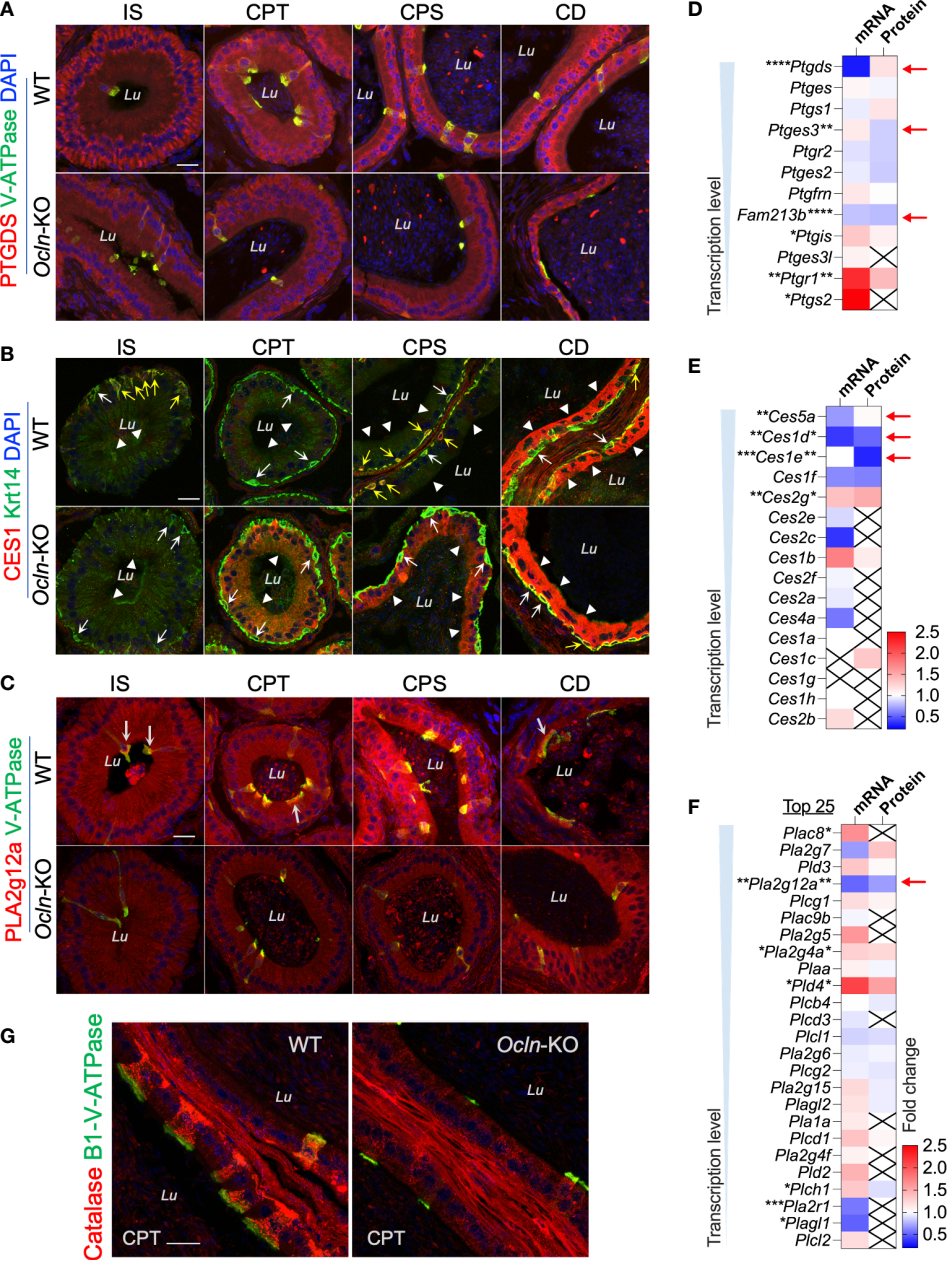
Expression levels of genes relating to lipid metabolism in the epididymis of *Ocln*-KO compared to WT mice. **(A)** Regional distribution and cellular localization of prostaglandin enzyme PTGDS in WT and *Ocln-*KO mouse epididymis. **(B)** Double-immunofluorescent labelling using a pan-CES1 antibody (red) with the basal cell marker Krt-14 (green) showing the regional distribution and cellular localization of CES1 in WT and *Ocln-*KO mouse epididymis. White arrows: basal cells with negligible CES1 labelling. Yellow arrows: basal cells with intense of CES1 immunofluorescent labelling. Arrowheads: principal cells. **(C)** Regional distribution and cellular localization of PLA2g12a in WT and *Ocln-*KO mouse epididymis. Arrows: enriched PLA2g12a in the apical domain of clear cells labeled in green for B1-V-ATPase. **(D-F)** Heatmaps of the DEGs relating to arachidonate-associated derivatives converting enzymes **(D)** and carboxylate esterases **(E)**, and membrane-associated or secreted phospholipases **(F)** in the transcriptomes and proteomes of mouse whole epididymis. Arrows indicated the genes significantly downregulated in the transcriptomes and/or proteomes. **(G)** Immunolabelling of epididymal cryosection showing enriched catalase in clear cells of WT epididymis and the obviously decreased expression in the clear cells of *Ocln-*KO epididymis. Lu, lumen; IS, initial segment; CPT, caput; CPS, corpus; CD, cauda epididymidis. Data were analyzed by two-tailed unpair t-test; *P< 0.05 and **P< 0.01, ***P< 0.001, ****P< 0.0001 (n>3 mice per group). Scale bar 20 µm.

**Figure 8:**
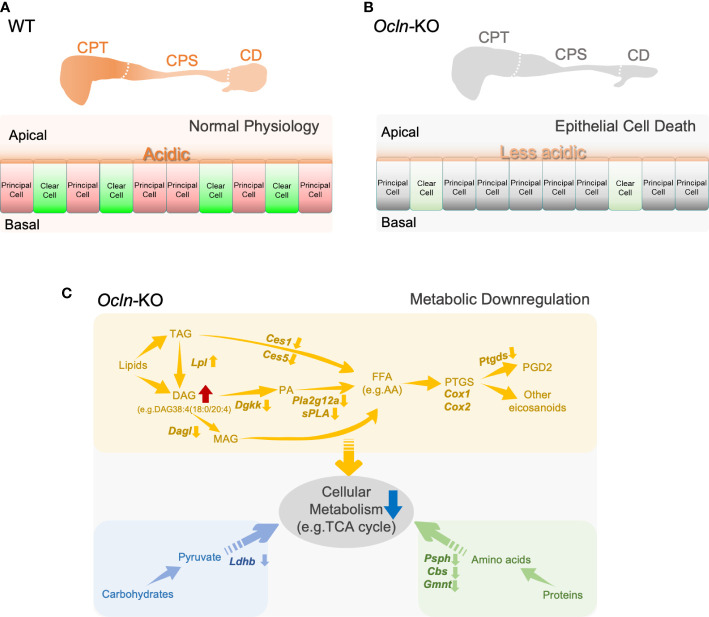
Schematic representation of a proposed model for OCLN-promoted acid-secretion and metabolic homeostasis and suggested a role of lipid catabolic enzymes in the mouse epididymis. **(A, B)** The proposed model of OCLN-promoted epithelial cells survival under normal physiological conditions in WT epididymis or impaired luminal acidification due to loss of clear cells under pathological conditions in *Ocln-*KO epididymis. **(C)** A schematic representation model illustrating the involvement of catabolic enzymes in metabolism in the epithelial cells especially the arachidonic fatty acid and eicosanoid pathways significantly altered in the *Ocln-*KO epididymis. AA, arachidonic acid; FFAs, free fatty acids; PTGS, prostaglandin-endoperoxide G/H synthase or COX1/COX2; GPLs, glycerolphospohlipids; PA, phosphatidic acid; LPA, lysophosphatidic acid; DAG, diacylglycerides; MAG, monoacylglycerols; TAG, triacylglycerides. See text for more details.

To determine the cellular localization of the shortlisted fatty acid catabolic enzymes, immunofluorescent labelling was applied on epididymal cryosections from WT and OCLN-deficient mice, respectively, for commercial antibodies directed against pan-CES1, PTGDS or PLA2g12a (**Figures 7A-C**). The antibody for proton-pump B1-V-ATPase was also used to label clear cells. The immunoreactivity for PTGDS was found in the endoplasmic network of principal cells of WT epididymis. It was found in some luminal contents in the distal epididymis. In addition, it was also enriched in the apical domain of V-ATPase-positive clear cells. In the OCLN-deficient epididymis, PTGDS in the principal cells was obviously decreased throughout the epididymal tubules (**Figure 7A**). Carboxylic esterase CES1 immunofluorescent labelling was enriched in the basal cells (**Figure 7B**, yellow arrows), where were marked with anti-keratin-14 antibody (**Figure 7B**, white arrows), as we published previously (29). From WT corpus to cauda regions, CES1 was highly abundant in the principal cells of cauda epididymidis (**Figure 7B**, arrowheads). In the OCLN-deficient epididymis, CES1 immunofluorescent intensity was increased moderately in the principal cells of caput and corpus epididymidis, but decreased in the basal cells marked with keratin-14 (**Figure 7B**, white and yellow arrows). The co-localization of CES1 and WT basal cells (**Figure 7B**, yellow arrows), especially in the WT corpus region, was obviously diminished in the *Ocln-*KO epididymis (**Figure 7B**, white arrows). As for PLA2g12a, in the WT epididymis, although its immunofluorescent intensity was obvious in the luminal contents, we consistently observed moderate intensity in the cytoplasm of epithelial cells, especial corpus region (**Figure 7C**). It was enriched levels in the apical domain of clear cells. In the *Ocln-*KO, PLA2g12a was obviously decreased in the cytoplasm of epithelial cells, but remained in the luminal contents (**Figure 7C**). Heatmap analysis on the transcriptomes and proteomes results showed that several related members were also significantly downregulated or altered in each of these pathways (**Figures 7D-F**, red arrows). The redox-sensitive metabolic-associated enzyme catalase was found to be enriched in clear cells of WT epididymis but significantly downregulated in the OCLN-deficient epididymis (**Figure 7G**). Based on all these results, we proposed a model for the OCLN-promoted metabolic pathways, especially lipid catabolism in the epididymal epithelial cells under physiological conditions and in pathological conditions after *Ocln* ablation, as shown in **Figure 8**. This involves impaired luminal acidification due to loss of clear cells (**Figures 8A, B**), as well as downregulated catabolic enzymes in DAG-related lipid metabolism and the subsequent arachidonate and eicosanoid pathways in *Ocln-*KO epididymis (**Figure 8C**).

## Discussion

In the epididymis, the epithelial cells themselves are not only responsible for the metabolism, but also nourish the spermatozoa maturing in the luminal microenvironment to keep spermatozoa in a quiescent state. One feature is the acidic luminal microenvironment maintained during spermatozoa transiting and undergoing maturation in the epididymis. The present study shows that tight-junction OCLN, located at the apical junction membrane of principal cells of proximal epididymis, is involved in ensuring epididymal integrity, acid secretion, and cellular metabolism, thereby ensuring male fertility.

The role of OCLN in promoting lipid metabolism and acid secretion is in line with the general understanding that the final products of metabolism are carbon dioxide (CO_2_), energy (e.g. ATP) and water. In the epididymis, it has been known that both luminal acidification and regulation of metabolism play essential roles in sperm maturation, as illustrated in the proposed model in **Figure 8**. Both epididymal acidification and metabolic syndromes, especially lipid metabolism disorders, are associated with male reproductive defects and infertility (16, 19–21, 47–50). A common feature shared by metabolism and acid secretion is the regulation of acid-base balance. As in other biological systems, CO_2_ hydrolyses spontaneously in water to become bicarbonate, and CO_2_-bicarbonate equilibrium is an essential process contributing to acid-base balance in the epididymis (8, 16, 46). Together with pumps and transporters, including CO_2_-bicarbonate permeable anion channels in principal cells, such as CFTR (2, 4), the clear cells expressing abundant proton pump V-ATPase play an indispensable role in luminal acidification of epididymis (15, 16, 51, 52). The purinergic agonist ATP, presumably released from the epididymal principal cells through CFTR anion channel and fluid transporter AQP9 (53–56), and its metabolites liberated in the epididymal lumen, also contribute to acid-base balance by activating clear cells and promoting acid secretion (57, 58). Increased apoptotic epithelial cells in the lumen, accompanying decreased clear cell population in the late rather than early postnatal age of *Ocln-*KO epididymis suggested that clear cells are sensitive to the loss of OCLN. The loss of clear cells can be due to an increase of apoptosis and/or impaired cell proliferation. Our results showed that clear cells can still be differentiated at the early age, although impaired clear cell differentiation cannot be excluded. In addition, our results supported an increase of apoptosis or programmed cell death of clear cell from the prepubertal age during sperm arrival and throughout adulthood rather than earlier postnatal age. Our results also implied that clear cells might undergo programmed cell death through different mechanisms, which deserve further investigation. This phenomenon suggests the involvement of survival signal, such as arachidonate-signaling for clear cell maintenance. And prostaglandin PGD2 has been reported to regulate epididymal epithelial apoptosis (59), whereas OCLN ablation can lead to epithelial apoptosis (60). We speculate that this prostaglandin-mediated signaling pathway is essential after sperm arrive in the epididymis due to the increased metabolic load from spermatozoa. This scenario also explains the degenerative phenotype of distal epididymis in the OCLN-deficient adult mice. Confocal immunofluorescent imaging of epididymal cryosection detected OCLN located at the apical junctions of principal cells in the WT proximal epididymis, whereas degenerated tubular defects are often observed in the distal epididymis. These results suggested that OCLN also plays an essential role in the regulation of acid-base balance and cellular metabolism by involving long-distance signaling pathways across proximal to distal compartments of the epididymis, suggesting the role of OCLN in secretion (61, 62), as well as cell-to-cell crosstalk for the homeostatic regulation in the epididymis (4, 5, 8, 51).

To elucidate the signaling pathways involved in OCLN-promoted metabolism and acid-base balance, we employed omics-based bioinformatics analysis. The DEGs in OCLN-deficient epididymis revealed that the significantly downregulated genes are mostly the enzymes relating to metabolism. The top impaired pathways include those associated with lipid catabolism, especially the catabolic enzymes relating to arachidonic acid and its derivatives, eicosanoids, such as *Ptgds* and *Pla2g12a*. PTGDS is a lipocalin-type prostaglandin D synthase that catabolizes the arachidonic acid epoxide derivative prostaglandin H2 to PGD2 (63). PLA2g12a is a non-membrane-bound soluble form of PLA2, also known as lipoprotein‐associated PLA2, which acts both extracellularly and intracellularly (64, 65). It has been known that cyclooxygenase (COX, also known as prostaglandin-endoperoxide G/H synthase) and arachidonic acid-derived eicosanoid system play essential role in epididymal function and male reproduction (6, 66, 67), especially the prostaglandin D2 pathway in the epididymis (59, 68). Prostaglandins, the major family in eicosanoid system, are bioactive lipid mediators derived from phospholipid hydrolysis by combined activities of PLA2 and COX or lipoxygenases. Transcriptomes showed no obvious changes in COX1 expression, though COX2 was slightly increased, whereas the cellular immunofluorescent results showed altered cellular localization of COX1 from Golgi organelles in the WT cells to a pattern of dispersed throughout cytoplasm of *Ocln-*KO epithelial cells (**Supplementary Figure 5**). Cellular localization analysis showed that both PTGDS and PLA2g12a proteins, the enzymes relating to prostaglandin catabolism, were present in the intracellular cytoplasmic organelles of WT epididymal epithelial cells and sporadically in some large debris in the lumen. PTGDS was enriched in the endoplasmic organelles near peri-nuclear and basolateral domains of epididymal principal cells. PLA2g12a was abundant in the apical domain of clear cells and at a weak level in the cytoplasm of principal cells. In view of the oxidative labile property of epoxide derivative prostaglandin H2, the production of eicosanoids is determined by the sources of arachidonic acid and the sub-compartmentalized enzymes. Thus, the distinct cellular localization of PLA2g12a and PTGDS suggests that arachidonic acid and PGD2 exert their signals in distinct subcellular compartments of epididymal epithelial cells and in the luminal contents, which are perturbed in OCLN-deficient epididymis. In addition, the enriched expression of PLA2g12a and PTGDS in clear cells of WT epididymis but decreased in the OCLN-deficient epididymis are also consistent with the notion that clear cells also participate in lipid metabolism in the epididymis.

Fatty acid chains conjugated to DAG and/or TAG are an important source of arachidonic acid and thus are important upstream precursors of lipid-mediated signaling pathways. Arachidonic acid is a 20-carbon unsaturated fatty acid chain containing four double-bonds. Our results showed that impaired release of free long-chain fatty acids, such as arachidonic acid, from DAG is one of the major defects in the OCLN-deficient epididymis. This notion is supported by our lipidomic data, in which DAG38:5(16:0/22:5) with long fatty acid unsaturated chains in the proximal epididymis and arachidonate-conjugated DAG38:4(18:0/20:4) in the distal epididymis were significantly accumulated in the OCLN-deficient epididymis. In addition, the enzymes known to be involved in this process were all downregulated, including *Dagl, Dgkk* and *PLA2g12a*. Regarding TAG, under normal physiological conditions, our results showed that a large proportion of the TAG species containing the unsaturated 22-carbon fatty acid chain, TAG58:7(22:6), was converted to other forms in the distal versus proximal epididymis. However, this conversion remained effective in the *Ocln-*null epididymis, suggesting that releasing unsaturated long fatty acid chains from TAG58:7(22:6) was independent of the loss of OCLN and/or there is a compensation conversion pathway upregulated. Consistent with both speculations, the content of TAG58:7(22:6) in the caput epididymidis was slightly decreased, indicating a net reduction in the synthesis of this TAG component, and triglyceride lipase *Lpl* expression was slightly upregulated in *Ocln-*null epididymis.

In addition to the PLA2 lipid-releasing enzymes, our omics results also suggested that the ester lipase carboxylic esterase enzymes, including CES1 and CES5, may also be involved in releasing fatty acid acyl chain from esters dependent on the function of OCLN. It has been known that CES1 enzymes can hydrolyze fatty-acid chains from conjugated lipids, including DAG and TAG and cholesteryl esters (45, 69). We speculate that CES is essential for cholesteryl ester hydrolysis, and if the arachidonic acid is the acyl link in the cholesteryl esters (45), then it also affects the arachidonic acid downstream pathways, causing the defective phenotype as in *Ocln-*KO. Our immunofluorescent labelling results showed that, in the WT epididymis, CES1 is predominantly expressed in basal cells from proximal to distal regions, and only in principal cells in distal regions. However, CES1 was significantly upregulated in principal cells but decreased in basal cells of caput epididymidis of OCLN-deficient mice. A potential role for basal cells in epididymal lipid homeostasis has been reported (70). The altered cellular localization of CES1 enzyme in basal cells and principal cells after the *Ocln* ablation deserves further investigation.

In summary, we have demonstrated that tight-junction protein OCLN promotes cellular metabolism, especially fatty acid catabolism and signaling, maintains the survival of proton-pump V-ATPase expressing clear cells and thereby acidity of the lumen, and ensures epididymal integrity for sperm maturation and male fertility. Notably, the cellular localization of OCLN in the apical junctional membrane of epididymal principal cells suggests that OCLN may exert its function through regulation membrane-related activities. Impaired acid-base balance, cellular metabolism and intracellular communication are also consistent with the involvement of OCLN in epithelial transport processes to maintain epididymal homeostasis. Epithelial transport involves extensive membrane events and the role of OCLN-dependent membrane-associated activities in epididymal cells remains unclear and deserves further investigation.

## Data availability statement

The original contributions presented in the study are publicly available. This data can be found here: The RNA-sequencing data have been deposited in NCBI's Gene Expression Omnibus and are accessible through GEO series accession number GSE166141 (https://www.ncbi.nlm.nih.gov/geo/query/acc.cgi?acc=GSE166141). The mass spectrometry proteomics data have been deposited to the ProteomeXchange Consortium via the iProX partner repository, with the dataset identifier PXD037690 (http://proteomecentral.proteomexchange.org/cgi/GetDataset?ID=PXD037690).

## Ethics statement

All animal experiments were approved by the Institutional Animal Care and Use Committee (IACUC) of ShanghaiTech University. Written informed consent was obtained from the owners for the participation of their animals in this study.

## Author contributions

WS conceptualized, experimental designed, data analysis, curation, and interpretation. WS and BL wrote the manuscript. BL and BZ performed most of the experiments. DG performed the animal phenotypic analysis as well as some omics and imaging experiments. XX performed some imaging experiments. QL participated in experiments of IVF and ICSI experiments. All authors contributed to the article and approved the submitted version.

## Funding

This work was financially supported by the National Natural Science Foundation of China (NNSFC 31871166, 82071704), and ShanghaiTech University.

## Acknowledgments

The authors thank all the staff of ShanghaiTech core facilities for their technical support, especially Dr. Xiao-Ming Li of Imaging Core Facility Dr Pi-Liang Hao of Mass Spectrometry Core Facility and Dr Ying Xiong of Cell & Molecular Core Facility.

## Conflict of interest

The authors declare that the research was conducted in the absence of any commercial or financial relationships that could be construed as a potential conflict of interest.

## Publisher’s note

All claims expressed in this article are solely those of the authors and do not necessarily represent those of their affiliated organizations, or those of the publisher, the editors and the reviewers. Any product that may be evaluated in this article, or claim that may be made by its manufacturer, is not guaranteed or endorsed by the publisher.

## Supplementary material

Supplementary Figure 1
**(A)** H&E staining, γH2AX immunofluorescence **(B)** and phalloidin **(C)** staining showed normal anatomical structure and spermatogenesis in the testes of young adult mice. **(D)** Retrieval of next-morning copulation plug shows fewer *Ocln-*KO sperm in deposition in female tract compared to WT male control. **(E)** ICSI assay results using cauda epididymidal spermatozoa collected from WT or *Ocln-*KO mice. **(F)** Normal pups from the ICSI-fertilized eggs with either WT or *Ocln-*KO cauda sperm.

Supplementary Figure 2 Immunofluorescent double-labelling for B1-V-ATPase and TUNEL staining of the different regions of 5-weeks old epididymis revealed a significantly raised apoptotic index in the *Ocln-*KO compared to WT mice. Yellow triangles: examples for TUNEL-positive cellular debris also showed some positive labelling of the clear cell marker B1-V-ATPase at weak levels. White triangles: B1-V-ATPase-positive but TUNEL-negative cells or debris in the lumen of caput epididymis. Blue: DNA stained with DAPI. Lu: lumen. Arrows: non-B1-V-ATPase-positive somatic cells in the lumen. Asterisks: TUNEL-positive sperm in the lumen characterized by their oval shaped heads. Scale bar: 20 µm.

Supplementary Figure 3 Principal components analysis of lipidomic data showing the differences of the components in different groups. **(A)** The lipidomic data are divided into 10 dimensions according to the percentage of explained variances method. **(B)** The lipid species with the top ten contributions in dimension-1. **(C)** The lipid species with the top ten contributions in dimension-2. Principal component analysis (PCA) is a widely used tool for dimensionality reduction and feature extraction in the field of omics data analysis. **(D)** The component clustering of different groups between dimension1 and dimension2 according to Zero-centered method. As clearly visible, good separation of WT proximal epididymis (WT-Proximal) versus distal epididymis (WT-Distal), and *Ocln-*KO proximal epididymis (KO-Proximal) versus distal epididymis (KO-Distal) clusters were achieved.

Supplementary Figure 4 Heatmap showing relative contents of TAG components in the proximal epididymis of OCLN-deficient mice. (A) Heatmaps showing the variation of the mean of row-normalized contents of TAG compositions in the proximal epididymis of WT and *Ocln-*KO mice. (B) Heatmaps showing the variation of row-normalized contents of TAG compositions in the distal epididymis of WT and *Ocln-*KO mice.

Supplementary Figure 5 Immunofluorescent labelling of cryosections for pro-inflammatory marker COX1 (green) in WT and *Ocln-*KO epididymis. The enlarged image showing the infiltrated sperm with their DNA in the head region (arrows) and some immunological cells (asterisks) in the *Ocln-*KO CPT-CPS junctional interstitial space.
