# Supplementary figures and images for: Epididymal epithelial degeneration and lipid metabolism impairment account for male infertility in occludin knockout mice
Source: Front Endocrinol (Lausanne). 2022 Nov 28;13:1069319. doi: 10.3389/fendo.2022.1069319 (PMC9742356; doi:10.3389/fendo.2022.1069319)

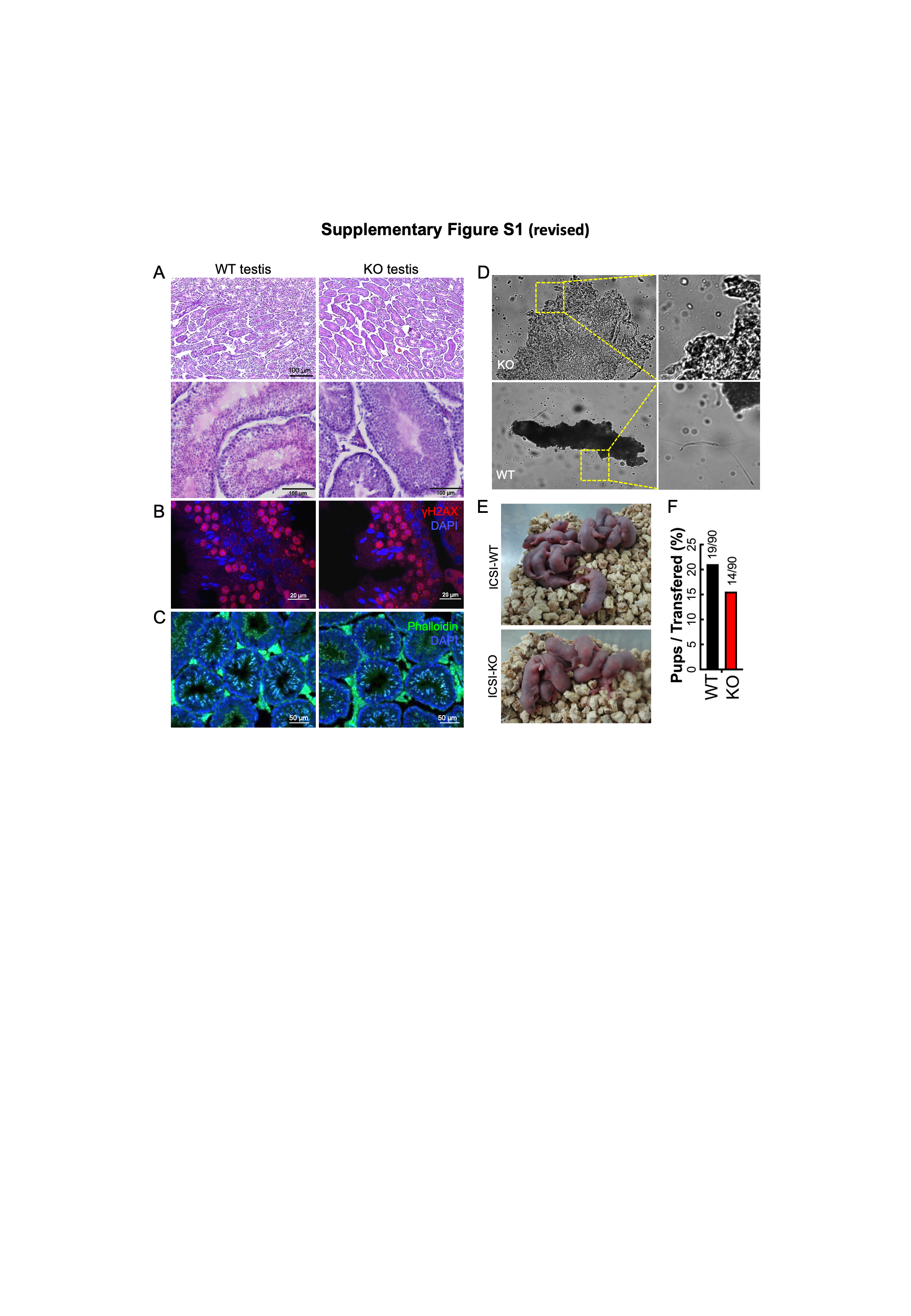

Supplement: Supplementary Figure 1 — (A) H&E staining, γH2AX immunofluorescence (B) and phalloidin (C) staining showed normal anatomical structure and spermatogenesis in the testes of young adult mice. (D) Retrieval of next-morning copulation plug shows fewer Ocln-KO sperm in deposition in female tract compared to WT male control. (E) ICSI assay results using cauda epididymidal spermatozoa collected from WT or Ocln-KO mice. (F) Normal pups from the ICSI-fertilized eggs with either WT or Ocln-KO cauda sperm. [file Image_1.tiff]

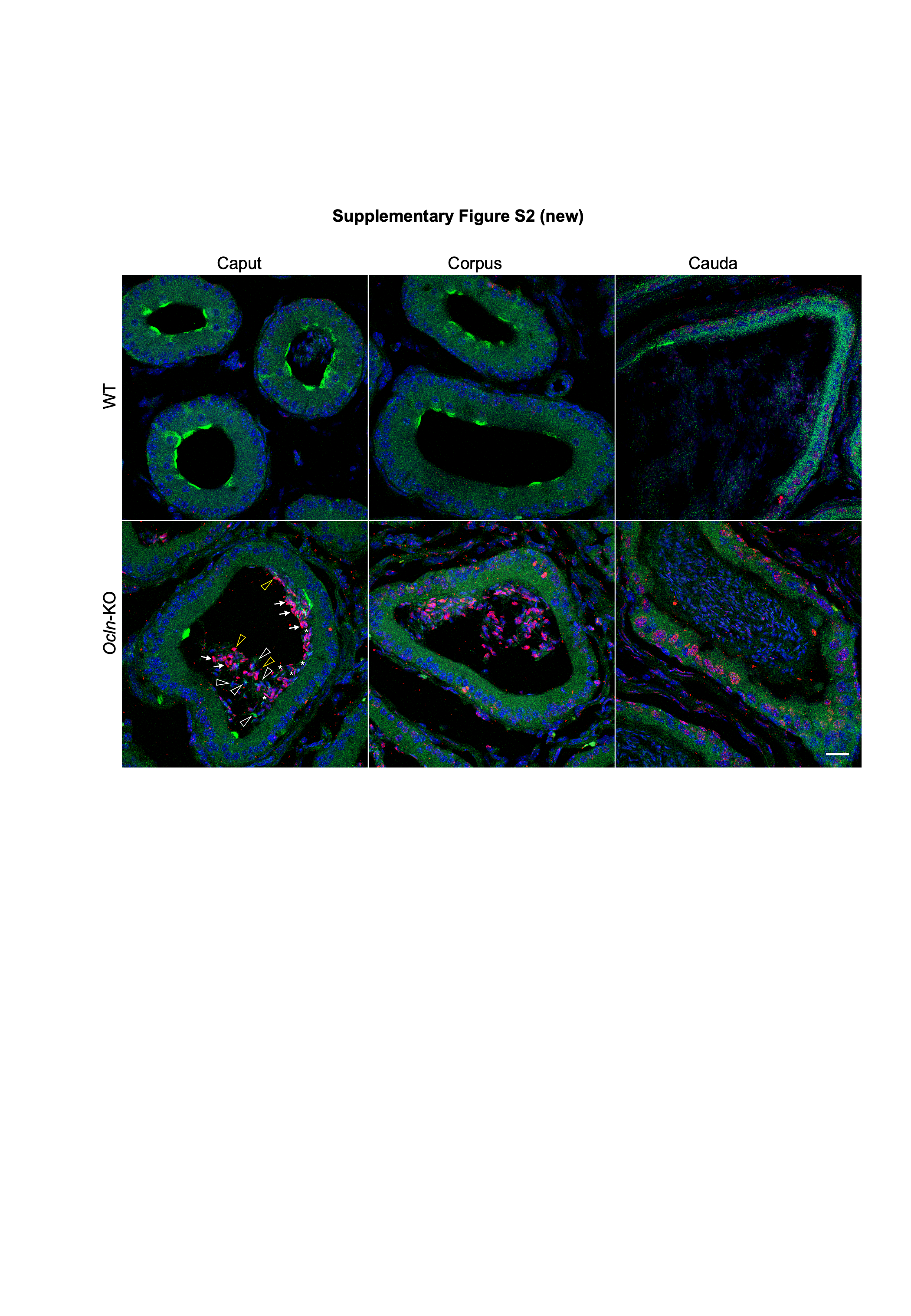

Supplement: Supplementary Figure 2 — Immunofluorescent double-labelling for B1-V-ATPase and TUNEL staining of the different regions of 5-weeks old epididymis revealed a significantly raised apoptotic index in the Ocln-KO compared to WT mice. Yellow triangles: examples for TUNEL-positive cellular debris also showed some positive labelling of the clear cell marker B1-V-ATPase at weak levels. White triangles: B1-V-ATPase-positive but TUNEL-negative cells or debris in the lumen of caput epididymis. Blue: DNA stained with DAPI. Lu: lumen. Arrows: non-B1-V-ATPase-positive somatic cells in the lumen. Asterisks: TUNEL-positive sperm in the lumen characterized by their oval shaped heads. Scale bar: 20 µm. [file Image_2.tiff]

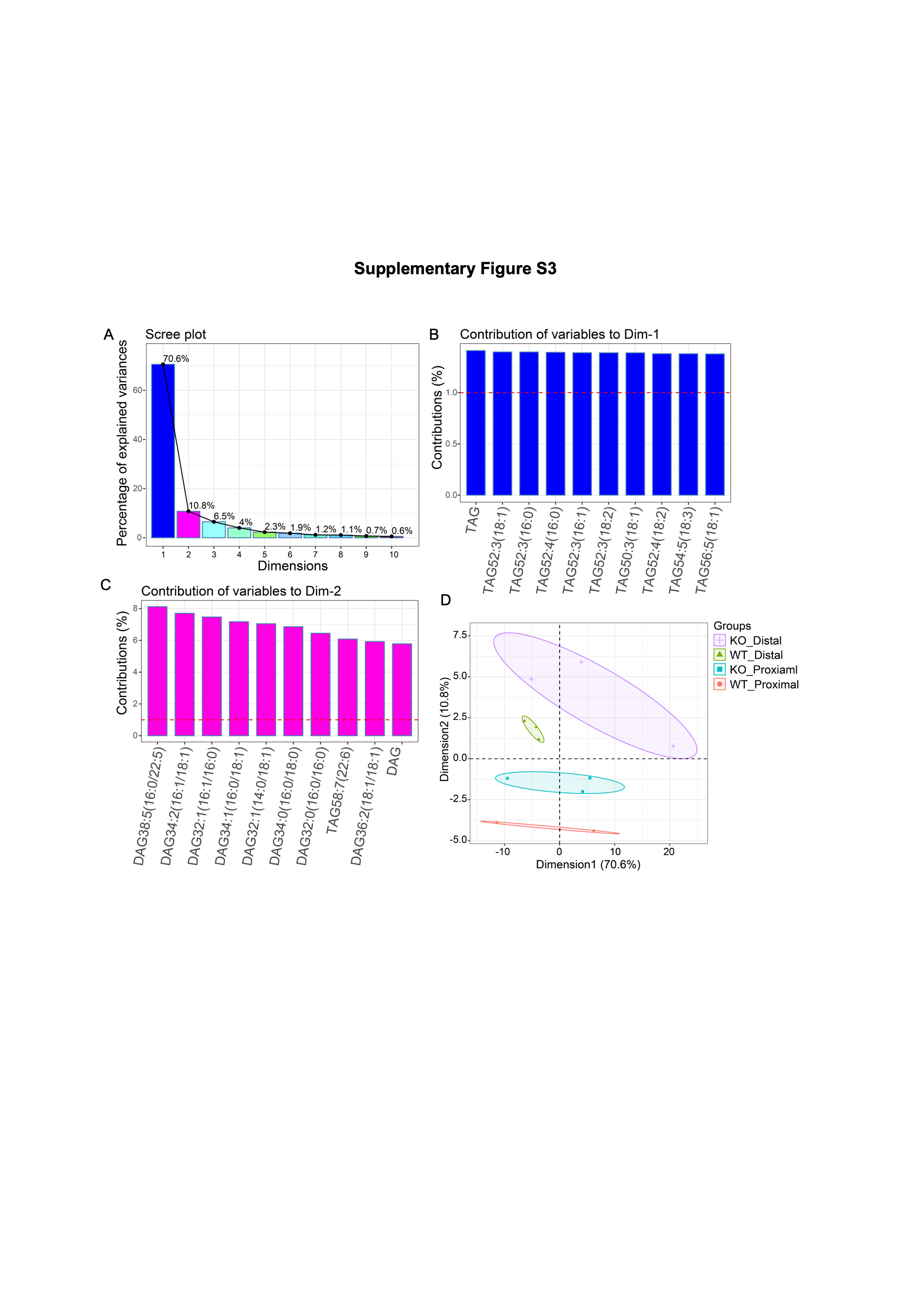

Supplement: Supplementary Figure 3 — Principal components analysis of lipidomic data showing the differences of the components in different groups. (A) The lipidomic data are divided into 10 dimensions according to the percentage of explained variances method. (B) The lipid species with the top ten contributions in dimension-1. (C) The lipid species with the top ten contributions in dimension-2. Principal component analysis (PCA) is a widely used tool for dimensionality reduction and feature extraction in the field of omics data analysis. (D) The component clustering of different groups between dimension1 and dimension2 according to Zero-centered method. As clearly visible, good separation of WT proximal epididymis (WT-Proximal) versus distal epididymis (WT-Distal), and Ocln-KO proximal epididymis (KO-Proximal) versus distal epididymis (KO-Distal) clusters were achieved. [file Image_3.tiff]

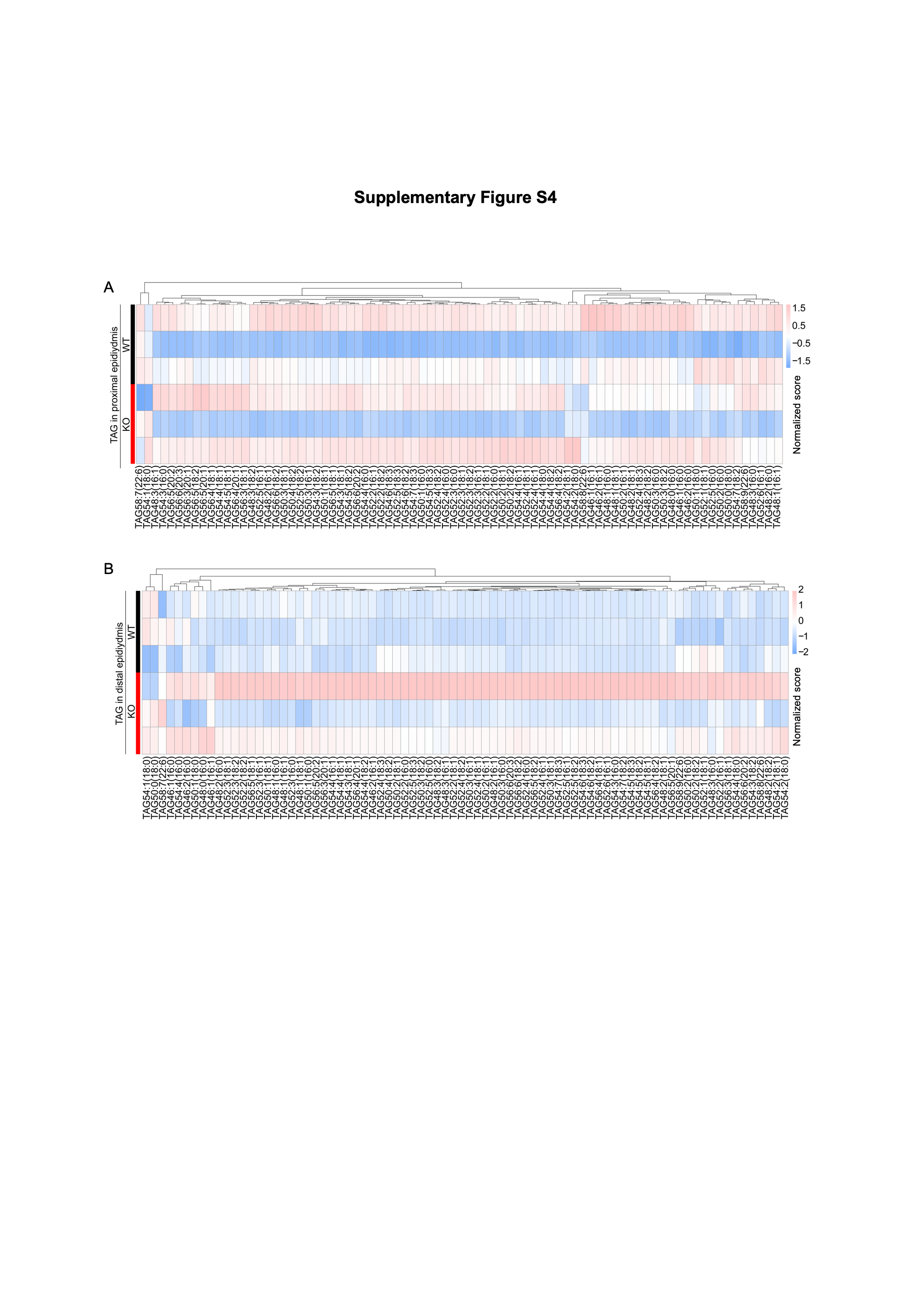

Supplement: Supplementary Figure 4 — Heatmap showing relative contents of TAG components in the proximal epididymis of OCLN-deficient mice. (A) Heatmaps showing the variation of the mean of row-normalized contents of TAG compositions in the proximal epididymis of WT and Ocln-KO mice. (B) Heatmaps showing the variation of row-normalized contents of TAG compositions in the distal epididymis of WT and Ocln-KO mice. [file Image_4.tiff]

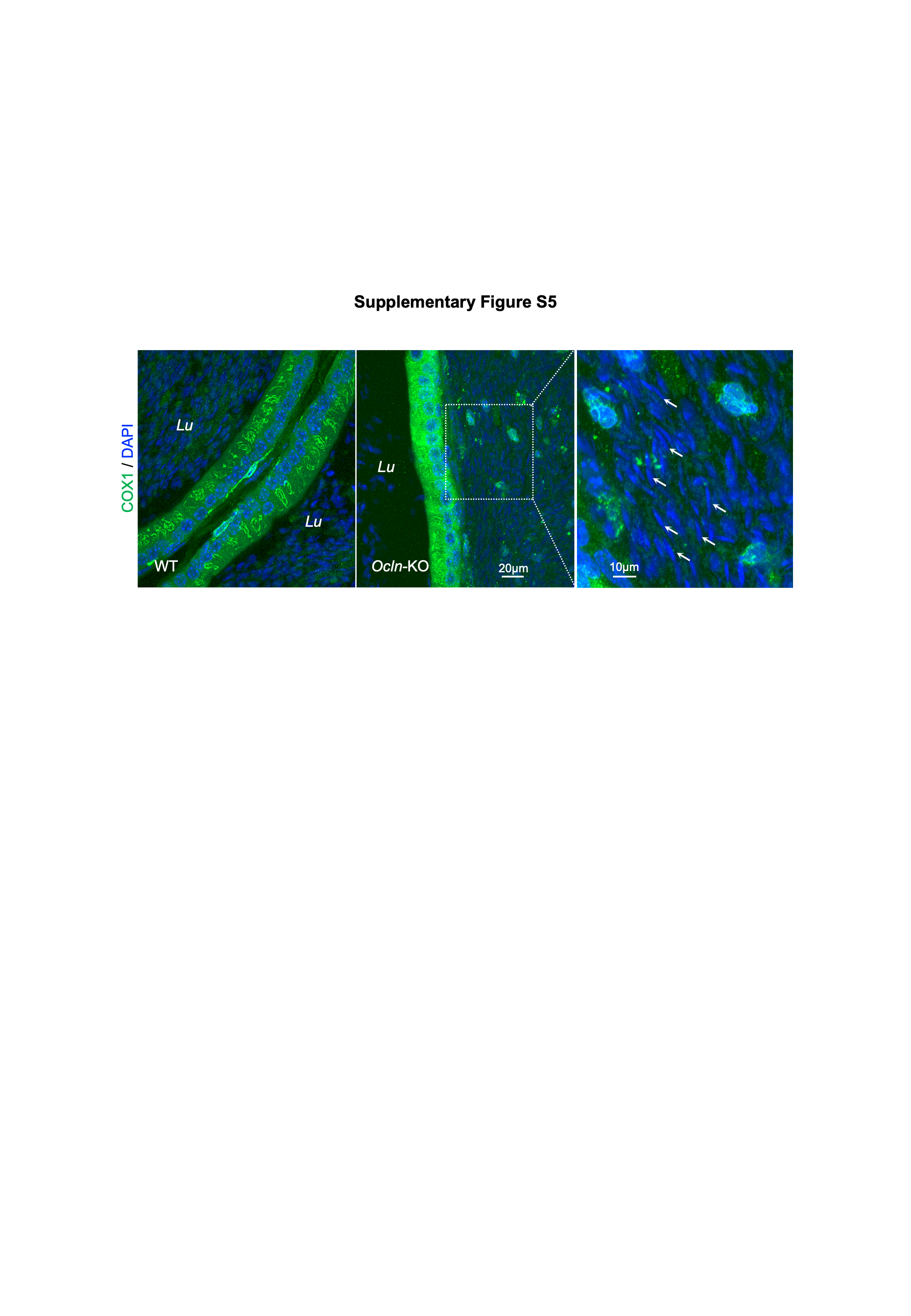

Supplement: Supplementary Figure 5 — Immunofluorescent labelling of cryosections for pro-inflammatory marker COX1 (green) in WT and Ocln-KO epididymis. The enlarged image showing the infiltrated sperm with their DNA in the head region (arrows) and some immunological cells (asterisks) in the Ocln-KO CPT-CPS junctional interstitial space. [file Image_5.tiff]
