# Supplementary material for: Epididymal epithelial degeneration and lipid metabolism impairment account for male infertility in occludin knockout mice
Source: Front Endocrinol (Lausanne). 2022 Nov 28;13:1069319. doi: 10.3389/fendo.2022.1069319 (PMC9742356; doi:10.3389/fendo.2022.1069319)
Supplement: Supplementary file 6 [file Table_1.docx]

Supplementary Table 1: List of Antibodies used in this study.

| **Antibody** | **Raised in** | **Source** | **Catalog** | **Work ConC.** |
| --- | --- | --- | --- | --- |
| Anti-Occludin | Rabbit | Gift from professor Breton |  | IF 1:100;  WB 1:2000 |
| Anti-B1-VATPase | Chicken | Gift from professor Breton |  | IF 1:60 |
| Anti-PLA2g12a | Rabbit | Absci | AB43382 | IF 1:200 |
| Anti-CK14 | Chicken | Millipore | MAB3232 | IF 1:200 |
| Anti-Keratin 18 | Mouse | Biolegend | 617201 | IF 1:100 |
| Anti-γH2AX | Rabbit | CST | 2577S | IF 1:100 |
| Anti-Actin | Mouse | Sigma | A5441 | WB 1:100000 |
| Anti-Catalase | Mouse | Santa Cruz | sc-271803 | IF 1:100 |
| Anti-Prostaglandin D Synthase | Rabbit | Abcam | ab182141 | IF 1:100 |
| Anti-CES1 | Rabbit | antibodies-online | ABIN5708325 | IF 1:100 |
| Anti-COX-1 | Mouse | Cayman Chemical | 160110 | IF 1:100 |
| FITC, anti-chicken antibody | Donkey | Jackson | 703-095-155 | IF 1:200 |
| FITC, anti-rabbit antibody | Donkey | Jackson | 111-095-144 | IF 1:200 |
| Cy3, anti-chicken antibody | Donkey | Jackson | 03-165-155 | IF 1:800 |
| Cy3, anti-rabbit antibody | Donkey | Jackson | 711-165-152 | IF 1:800 |
| Cy3, anti-mouse antibody | Donkey | Jackson | 715-165-150 | IF 1:800 |
| HRP, anti-mouse | Donkey | Jackson | 715-036-151 | WB 1:100000 |
| HRP, anti-rabbit | Donkey | Jackson | 711-036-152 | WB 1:100000 |

Note: IF: immunofluorescent labelling; WB: western blotting analysis.
